# 2527 Mount Sinai health hackathon: Harnessing the power of collaboration to advance experiential team science education

**DOI:** 10.1017/cts.2018.218

**Published:** 2018-11-21

**Authors:** Janice Lynn Gabrilove, Peter Backeris, Louise Lammers, Anthony Costa, Layla Fattah, Caroline Eden, Jason Rogers, Kevin Costa

**Affiliations:** Icahn School of Medicine at Mount Sinai

## Abstract

OBJECTIVES/SPECIFIC AIMS: Innovation in healthcare is increasingly dependent on technology and teamwork, requiring effective collaboration between disciplines. Through an intensive team-based competition event, Mount Sinai Health Hackathon 2017, aimed to harness the power of multidisciplinary and transdisciplinary collaboration to foster innovation in the field of cancer. Participants were immersed in an intensive weekend working in teams to develop technology solutions to important problems affecting patients and care providers in the field of cancer. The learning objectives were to enable participants to: Identify cancer-related healthcare problems which lend themselves to technology-based solutions. Delineate key behaviors critical to multidisciplinary team success Identify optimal strategies for communicating in multidisciplinary teams. Engage and inspire participants to apply knowledge of technology to meaningfully impact clinical care and well-being. METHODS/STUDY POPULATION: The Mount Sinai Health Hackathon is an annual 48-hour team-based competition, using a format adapted from guidelines provided by MIT Hacking Medicine. The 2017 event gathered a total of 87 participants (120 registered), representing 17 organizations from as far away as California, with a diverse range of backgrounds in bioinformatics, software and hardware, product design, business, digital health and clinical practice. The overall participation model included: Phase 0: Health Hackathon 101 summer workshops; Phase 1: pre-Hackathon priming activities using online forums Trello and Slack; Phase 2: a 48-hour onsite hackathon to catalyze innovation through problem sharing, solution pitches, team formation and development of prototype solutions; Phase 3: competitive presentations to judges and prize awards; Phase 4: a suite of post-hackathon support to stimulate continued development of innovations. The event sponsored by ConduITS, was also co-sponsored by Persistent Systems, IBM Watson, Tisch Cancer Institute, Sinai AppLab, Sinai Biodesign and other ISMMS Institutes. Mentors circulated throughout the event to support the teams in the technical, clinical, and business development aspects of their solutions. In total, the 14 teams formed during the Hackathon, created innovations ranging from diagnostic devices, networking apps, artificial intelligence tools, and others. The top 3 teams were each awarded $2500 to support their projects’ future development. RESULTS/ANTICIPATED RESULTS: Qualitative and quantitative post-event survey data revealed the Hackathon experience fostered collaborative attitudes and a positive experience for participants, providing insight into the potential benefits of team science. In the post-event survey (n=24) 92% of participants reported that the experience increased their ability to solve problems and 96% made new professional or personal connections. In addition, 96% of respondents would attend future Hackathon events and 75% reported they were likely to continue working on their project after the Hackathon. Qualitative feedback from 1 participant reported it was: “a wonderful event that really highlighted how much interdisciplinary team science can achieve.” Along with intermediate support interactions, including the winning teams participating in a Shark Tank style event with pitches to external entrepreneurs and investors, all teams will be followed up in 6 months time to determine if participants continue to work on projects, file new patents, create new companies, or leverage the new connections made through the Health Hackathon experience. DISCUSSION/SIGNIFICANCE OF IMPACT: Our experience indicates that a Health Hackathon is a compelling and productive forum to bring together students, trainees, faculty, and other stakeholders to explore tech-based solutions to problems in cancer and other areas of biomedicine. It is a valuable tool to foster collaboration and transdisciplinary team science and education. Follow-up analysis will determine to what extent the Mount Sinai Health Hackathon is contributing to an ecosystem that encourages professionals and trainees in healthcare and in technology development to work together to address unmet needs in healthcare with innovative technology solutions.

